# Impact of a Virtual Care Navigation Service on Member-Reported Outcomes Among Lesbian, Gay, Bisexual, Transgender, and Queer Populations: Case Study

**DOI:** 10.2196/64137

**Published:** 2025-01-09

**Authors:** Seul Ki Choi, Jaclyn Marshall, Patrina Sexton Topper, Andrew Pregnall, José Bauermeister

**Affiliations:** 1 School of Nursing, University of Pennsylvania, 418 Curie Blvd, Philadelphia, PA, 19104, United States, 1 8123695216; 2 Included Health, San Francisco, CA, United States; 3 College of Nursing, Villanova University, Villanova, PA, United States; 4 Perelman School of Medicine, University of Pennsylvania, Philadelphia, PA, United States

**Keywords:** health care navigation, LGBTQ+, lesbian, gay, bisexual, transgender, queer, access to care, care avoidance, identity-affirming care, minority, stress, stigma, health outcomes

## Abstract

**Background:**

While the significance of care navigation in facilitating access to health care within the lesbian, gay, bisexual, transgender, queer, and other (LGBTQ+) communities has been acknowledged, there is limited research examining how care navigation influences an individual’s ability to understand and access the care they need in real-world settings. By analyzing private sector data, we can bridge the gap between theoretical research findings and practical applications, ultimately informing both business strategies and public policy with evidence grounded in real-world efficacy.

**Objective:**

The objective of this study was to evaluate the impact of specialized virtual care navigation services on LGBTQ+ individuals’ ability to comprehend and access necessary care within a national cohort of commercially insured members.

**Methods:**

This case study is based on the experience of commercially insured members, aged 18 or older, who used the LGBTQ+ Health Care Navigation (LGBTQ+ Navigation) service by Included Health between January 26 and July 31, 2023. Care coordinators assisted members by connecting them with vetted identity-affirming in-network providers, helping them navigate and understand their LGBTQ+ health benefits, and providing education and advocacy for clinical and nonclinical needs. We examined the impact of navigation on 5 member-reported outcomes. In addition to reporting the proportion who agreed or strongly agreed, we calculated an impact score that averaged assigned numerical values to all 5 question responses (1=strongly disagree to 5=strongly agree) for each respondent. We used ANOVA with Tukey post hoc tests and *t* tests to explore the relationships between the impact score and member characteristics, including optional self-reported demographics.

**Results:**

Out of 4703 LGBTQ+ Navigation cases, 7.53% (n=354) had member-reported outcomes. A large majority of LGBTQ+ members agreed or strongly agreed that care navigation resulted in less stress (315/354, 89%), less care avoidance (305/354, 86.2%), higher confidence in finding an identity-affirming provider (327/354, 92.4%), improved ability to comprehend health care information (312/354, 88.1%), and improved ability to engage with providers (308/354, 87%). The average impact score was 4.44 (SD 0.69), with statistically significant differences by gender identity (*P*=.003), race (*P*=.01), ethnicity (*P*=.008), and pronouns (*P*=.02). The scores were highest for members with multiple gender identities (mean 4.56, SD 0.37), and members who did not provide their race, ethnicity, or their pronouns (mean 4.55, SD 0.64). Impact scores were lowest for transgender members (mean 4.11, SD 0.95).

**Conclusions:**

The LGBTQ+ Navigation service, by enhancing members’ comprehension and use of necessary care, demonstrates potential public health utility and value. Continuous evaluation of navigation services can serve as a supplementary tool for employers seeking to promote health equity and improve belonging among employees. This is particularly important as discrimination and stigma against LGBTQ+ communities persist in the United States. Therefore, scalable and system-level changes that use navigation services are essential to reach a larger proportion of the LGBTQ+ population.

## Introduction

Compelling evidence indicates that communities comprised of lesbian, gay, bisexual, transgender, queer, and a broader spectrum of sexual orientations and/or gender identities (LGBTQ+) disproportionately endure a range of health issues [1,3]. According to minority stress theory, the stigma associated with sexual and/or gender minority identities acts as a stressor, potentially serving as a foundational contributor to health inequities among LGBTQ+ people [4]. This inequity includes adverse health outcomes, such as various forms of cancer, mental health disorders including depression and anxiety, HIV, and higher rates of substance use compared to their heterosexual and cisgender counterparts [5,10].

Research has identified impeded access to health care services as a key driver of adverse health outcomes within the LGBTQ+ communities, further amplifying health inequities [11,13]. Drawing upon the comprehensive framework for health care access of Levesque et al [14], it is imperative to examine health care accessibility from a multifaceted perspective. This perspective can involve accounting for factors like lack of inclusive policies, limited availability of health insurance, financial challenges that make health care unaffordable, insufficient awareness of available health care services, a sense of mistrust toward health care providers, and poor quality health care related to discrimination based on sexual orientation and gender identity, as well as a lack of health care providers’ understanding of LGBTQ+ health care needs [15,24].

Policies and interventions that take this multidimensional view of health care access [14] are essential for improving access to health care, as well as improving access to the evidence-based medical information, all of which play a significant role in facilitating health care access [25]. One such intervention is care navigation, acknowledged as a successful model for increasing access to quality health care, particularly for marginalized populations [26]. Recently, small studies have demonstrated the ability of patient navigation to connect and engage individuals within diverse LGBTQ+ communities with HIV care [27] and facilitate gender-affirming care for transgender populations [28,29].

While the significance of care navigation in facilitating access to care within the LGBTQ+ communities has been acknowledged [27-29], research in this area is limited. One gap that remains is understanding how care navigation influences an individual’s ability to understand and access the care they need in real-world settings. Evaluating these services that are delivered by the private sector offers unique value that complements traditional research studies by providing real-world, actionable insights. Unlike controlled environments in research, private sector evaluations reflect the complexities of actual consumer behaviors, operational challenges, and market conditions. This allows for the observation of how services perform in diverse, dynamic settings where human behavior is less predictable and influenced by a range of contextual factors. Moreover, private sector data often encompasses larger, more varied populations, offering a broader scope of generalizability. By analyzing this data, we can bridge the gap between theoretical research findings and practical applications, ultimately informing both business strategies and public policy with evidence grounded in real-world efficacy. Therefore, the objective of the study was to evaluate the use of an LGBTQ+-specialized virtual care navigation service among Included Health’s members and assess the impact of these services on members’ abilities to understand and access the care they need among a national, commercially-insured cohort.

## Methods

This study follows STROBE (Strengthening the Reporting of Observational Studies in Epidemiology) reporting guidelines for cohort studies.

### LGBTQ+ Virtual Health Care Navigation Service

This study is based on the experience of commercially insured members, aged 18 or older, who used the LGBTQ+ Health Care Navigation (LGBTQ+ Navigation) service by Included Health. LGBTQ+ Navigation is Included Health’s phone- and chat-based health care navigation service for LGBTQ+ communities. It is offered by self-insured employers as a health care benefit for employees and their dependents and by health plans for their insured members. The LGBTQ+ Navigation service is consistent across all members who are eligible for the service. Employers share information about the navigation service with their employees and their family members similarly to other health benefits, which may include mailings, webinars, and their intranet. Members then proactively initiate a case by completing an intake form on Included Health’s website or within the digital health app. A team of care coordinators, 90% of whom identify as LGBTQ+, provide concierge advocacy and guidance for members across 3 areas: connections to vetted identity-affirming in-network providers, benefits navigation focused on LGBTQ+ health, and education and advocacy for clinical and nonclinical needs.

These providers, ranging from primary care physicians to specialists, have been vetted by Included Health to ensure familiarity with LGBTQ+ health needs and inclusive practices. Coordinators, trained in investigative interviewing, gather information by speaking directly to providers and their staff on the provider’s experience with population-level health needs, the treatment of LGBTQ+ members, and confirmation of inclusive practices, such as using the correct name and pronouns for patients regardless of their gender assigned at birth. If there are not multiple provider options for the member in the directory, coordinators conduct additional research and vetting to find suitable providers.

Included Health uses a rigorous vetting process to ensure that providers recommended for LGBTQ+ care offer a high standard of cultural competence and inclusivity. Rather than relying on binary yes or no questions or allowing providers to self-identify as inclusive, the care coordinators—who bring specialized training and lived experience—conduct in-depth conversations with providers. Care coordinators are trained over a 4 to 5 week program where they are prepared to address various topics including benefits plans, social determinants of health, family building, and common systemic health conditions faced by LGBTQ+ individuals. In addition, they receive training in patient advocacy and engage in intuitive listening practices to round out their deep practical knowledge with the skills to uncover needs and empower members. This approach allows coordinators to evaluate providers’ practical experience with LGBTQ+ communities and their commitment to delivering culturally affirming, high-quality care. To further ensure consistency in care standards, Included Health actively solicits feedback from LGBTQ+ members who visit these recommended providers. Providers who do not meet member expectations for culturally competent care are reviewed and, if necessary, removed from the directory. This continuous feedback loop, guided by both professional assessment and member experiences, helps to maintain a directory of providers who align with Included Health’s standards for LGBTQ+ inclusive care.

Care coordinators also assist members with understanding their benefits related to LGBTQ+ health, such as family expansion and gender-affirming care. For example, members interested in family expansion may have access to adoption support and reproductive endocrinology interventions. In addition, care coordinators assist the member in completing all the steps along gender-affirming care pathways, which may include any necessary documentation such as World Professional Association for Transgender Health (WPATH) letters and access to travel benefits if needed. They are trained to uncover additional needs, allowing them to connect members to benefits they might not know they have. In addition, the care coordinators support members through education and advocacy for clinical and nonclinical needs. This support may include helping the member navigate a claim denial, interpret the workplace policy guidance, identify community resources (eg, parents of transgender children support group), complete required paperwork prior to receiving care, and inform the member on how to change their legal name.

### Data Source

Our study sample consisted of all completed service requests (closed cases) of LGBTQ+ Navigation from January 26 to July 31, 2023. We merged data from member intake forms and the postcase member surveys. Members initiated one or more LGBTQ+ Navigation cases by completing an LGBTQ+ Navigation member request form. The form requested information to confirm LGBTQ+ Navigation eligibility, how the member could be supported (eg, find a provider or resource request), and geographic location, as well as optional fields for race, sexual orientation, pronouns, and gender identity. At case closure, the LGBTQ+-specialized care coordinator emailed an optional survey, without an incentive offered for completing the survey, to the member specific to the LGBTQ+ Navigation offering.

### Ethical Considerations

The University of Pennsylvania Institutional Review Board (IRB) reviewed the study’s protocol and concluded it was exempt as it did not meet the regulatory definition of human subjects research. The committee stated that “the IRB determined that this project qualifies as quality/program improvement meaning that the project does not meet the regulatory definition of human participants research and therefore does not require formal IRB review.” When submitting the form to request navigation services, the member agreed to the terms of service which included the Included Health privacy policy, which stated anonymous information may be used for research. Data were deidentified which ensured that the authors did not have access to any identifiable information about members. Compensation was not provided for study participation.

### Measures

#### Member and Case Characteristics

The member’s geographic region was defined as Midwest, Northeast, South, or West based on the US census regions. We also included the type of case request as a binary variable (a provider request or seeking resource/support) and whether the member had more than one case. Race, ethnicity, gender identity, sexual orientation, and pronouns were optional fields within the member intake form. The race and ethnicity question allowed respondents to select all that apply, aligning with US Census Bureau recommendations [30]. Due to limited sample size, we derived 2 variables: one indicating whether they identified as White and the other indicating whether they identified as Hispanic. For pronouns, individuals were presented with the option to select from he/him/his, she/her/hers, they/them/theirs, and ze/zir/zirs using a choose-all-that-apply format. Subsequently, we derived a variable indicating whether they chose a single set of pronouns or multiple pronouns.

In the original survey, participants could select all applicable options for gender identity and sexual orientation (see Supplement 2 in Multimedia Appendix 1) [31]. To create mutually exclusive variables for gender identity and sexual orientation, we compiled all the selected options and categorized them into distinct sexual orientations and gender identities. If respondents chose a single identity for either sexual orientation or gender identity, they were merged into a new variable informed by the National Academies’ Consensus Report [32]. For both gender identity and sexual orientation, some distinct identities had low sample sizes. In those instances, we combined the respondents into one category. If they selected multiple options for sexual orientation or gender identity, they were placed in a category for multiple options. Consequently, we established mutually exclusive categories for gender identity (cisgender; transgender; other gender identities including agender, gender fluid, intersex, or nonbinary; and multiple gender identities) and sexual orientation (bisexual; gay/lesbian; other sexual orientations including aromantic, asexual, pansexual, queer, or heterosexual; and multiple sexual orientations). For cases in which members did not complete the optional form fields, their member characteristics were presented as not available to indicate missing information. Details on the original responses for gender identity and sexual orientation, as well as how this data was merged into new mutually exclusive categories, can be found in the Supplement 2 in Multimedia Appendix 1.

#### Member-Reported Outcomes

Our study outcome examined members’ evaluation of LGBTQ+ Navigation using a 5-point scale (1=strongly disagree to 5=strongly agree). The questions began, “Since working with Included Health, I am…” and the 5 fragments were (1) less stressed about using health care services, (2) less likely to avoid care, (3) better able to find, understand, and use the information I need to inform my health care choices, (4) better able to find health care providers who understand my needs, and (5) more prepared to engage with health care providers.

In addition to the individual responses, we calculated a composite score for each member to denote the LGBTQ+ Navigation impact. Responses were averaged across the 5 questions (Cronbach α=0.94). A score of 4 or higher was considered an overall positive impact of LGBTQ+ Navigation, as it meant the respondent selected agree (4 points) or strongly agree (5 points) on average, across the 5 separate questions. A score less than 4 was considered a neutral to no impact of LGBTQ+ Navigation, since it indicated that the member was more likely to select neither agree or disagree, disagree, or strongly disagree across the questions.

### Statistical Analyses

We used descriptive statistics, including means and distributions, to report the study sample characteristics and the member-reported outcomes. To understand the differences between those with and without member-reported outcomes, we used Fisher exact tests or *χ*^*2*^ tests for categorical variables and 2-tailed *t* tests for continuous variables. Among the subset of individuals who completed a follow-up survey, we explored the relationships between the LGBTQ+ Navigation impact score and member characteristics. We used ANOVA with Tukey post hoc tests and *t* tests. The α level was defined as .05. All analyses were conducted in SAS version 9.4 (SAS Institute).

## Results

There were 4703 LGBTQ+ Navigation cases in the study sample, of which 354 (7.53%) cases were linked to postcase surveys with member-reported outcomes (Table 1). LGBTQ+ Navigation cases with member-reported outcomes were more likely to be for members with multiple cases (202/354, 57.1% vs 1761/4349, 40.49%; *P*<.001) and less likely to be from members who live in the South (98/354, 27.8% vs 1635/4349, 37.97%; *P*<.003) compared to cases without member-reported outcomes. Moreover, members who provided member-reported outcomes were less likely to provide their demographic characteristics, including race and ethnicity (178/354, 50.3% vs 1484/4349, 34.12%), pronouns (178/354, 50.3% vs 1484/4349, 34.12%), gender identity (205/354, 57.9% vs 1869/4349, 42.98%), and sexual orientation (190/354, 53.7% vs 1663/4349, 38.24%), in comparison to those who did not submit member-reported outcomes (*P*<.001). There was not a statistically significant difference in the proportion of cases that requested assistance in finding an identity-affirming provider (350/354, 98.9% vs 4229/4349, 97.24%; *P*=.07).

Among cases linked to member-reported outcomes, half of the respondents chose not to provide their race (178/354, 50.3%) and ethnicity (178/354, 50.3%), pronouns (178/354, 50.3%), gender identity (205/354, 57.9%), or sexual orientation (190/354, 53.7%). For the cases where race, ethnicity, and pronouns were provided (176/354, 49.7%), a majority identified as White (113/176, 64.2%) and non-Hispanic (141/176, 80.1%), and they used singular pronouns (149/176, 84.7%). For the cases where gender identities were provided (149/354, 42.1%), half of the members identified as cisgender (75/149, 50.3%), followed by transgender (33/149, 22.2%); other gender identities including agender, gender fluid, intersex, or nonbinary (30/149, 20.1%); or multiple gender identities (11/149, 7.4%). For the cases where sexual orientations were provided (164/354, 46.3%), 46.3% (76/164) identified as gay or lesbian, followed by multiple sexual orientations (40/164, 24.4%); other sexual orientations including aromantic, asexual, pansexual, queer, or heterosexual (30/164, 18.3%); or bisexual (18/164, 11%).

We found that a large majority of respondents agreed or strongly agreed that LGBTQ+ Navigation had a positive impact on their ability to understand and use the care they needed. Roughly 89% (315/354) felt less stressed about accessing health care services, and 86.2% (305/354) reported they were less likely to avoid care (Table 2). Furthermore, 88.1% (312/354) of respondents agreed or strongly agreed that they improved their ability to find, comprehend, and use the information necessary for making informed health care decisions. Over 92% (327/354) agreed or strongly agreed they had increased confidence in finding health care providers, and 87% (308/354) felt more prepared to engage with these providers.

The average composite LGBTQ+ Navigation impact score was 4.44 (SD=0.69). Approximately 44.6% (158/354) of members had a composite score of 5, indicating they responded “strongly agree” to each of the 5 statements. In addition, 36.2% (128/354) of scores were 4 or more but less than 5, while 19.2% (68/354) were less than 4 (Table 3).

The LGBTQ+ Navigation impact scores varied by case and member characteristics (Table 4). The mean impact scores were higher for members in the South and Midwest (South: 4.69; Midwest: 4.59; Northeast: 4.39; West: 4.20; *P*<.001); members with multiple gender identities (multiple gender identities: 4.56; not available: 4.55; cisgender: 4.36; other gender identities: 4.25; transgender: 4.11; *P*=.003); and members who did not provide their race (not available: 4.55; non-White: 4.40; White: 4.30; *P*=.01), their ethnicity (not available: 4.55; Hispanic: 4.46; non-Hispanic: 4.30; *P*=.008), or their pronouns (not available: 4.55; multiple pronouns 4.36; single set of pronouns: 4.33; *P*=.02). The impact scores did not differ significantly by sexual orientation.

**Table 1.** Associations between case and member characteristics and the completion status of member-reported outcomes (n=4703).

Variable	Total (n=4703), n (%)	Member-reported outcomes, n (%)		*P* value
		No (n=4349)	Yes (n=354)	
Region				.003
Midwest	694 (14.9)	635 (14.75)	59 (16.8)	
Northeast	809 (17.37)	738 (17.14)	71 (20.2)	
South	1733 (37.2)	1635 (37.97)	98 (27.8)	
West	1422 (30.53)	1298 (30.14)	124 (35.2)	
Requester service				.07
Provider request	4579 (97.36)	4229 (97.24)	350 (98.9)	
Resources/support	124 (2.64)	120 (2.76)	4 (1.1)	
Member had more than one case				<.001
No	2740 (58.26)	2588 (59.51)	152 (42.9)	
Yes	1963 (41.74)	1761 (40.49)	202 (57.1)	
Race				<.001
Non-White	1302 (27.68)	1239 (28.49)	63 (17.8)	
White	1739 (36.98)	1626 (37.39)	113 (31.9)	
N/A^a^	1662 (35.34)	1484 (34.12)	178 (50.3)	
Ethnicity				<.001
Non-Hispanic	2484 (52.82)	2343 (53.87)	141 (39.8)	
Hispanic	558 (11.86)	523 (12.03)	35 (9.9)	
N/A	1661 (35.52)	1483 (34.1)	178 (50.3)	
Pronouns				<.001
Single pronouns	2725 (57.94)	2576 (59.23)	149 (42.1)	
Multiple pronouns	316 (6.72)	289 (6.65)	27 (7.6)	
N/A	1662 (35.64)	1484 (34.12)	178 (50.3)	
Gender identity				<.001
N/A	2074 (44.1)	1869 (42.98)	205 (57.9)	
Cisgender	1677 (35.66)	1602 (36.84)	75 (21.2)	
Multiple gender identities	86 (1.83)	75 (1.72)	11 (3.1)	
Other^b^	422 (8.97)	392 (9.01)	30 (8.5)	
Transgender	444 (9.44)	411 (9.45)	33 (9.3)	
Sexual orientation				<.001
N/A	1853 (39.4)	1663 (38.24)	190 (53.7)	
Bisexual	276 (5.87)	258 (5.93)	18 (5.1)	
Gay/lesbian	1402 (29.81)	1326 (30.49)	76 (21.5)	
Multiple sexual orientations	497 (10.57)	457 (10.51)	40 (11.3)	
Other^c^	675 (14.35)	645 (14.83)	30 (8.5)	

a N/A: not available.

b Other gender identities included agender, gender fluid, intersex, or nonbinary.

c Other sexual orientations included aromantic, asexual, pansexual, queer, or heterosexual.

**Table 2.** Unadjusted average and distribution of responses by member-reported outcomes (n=354).

Response item	Response score, mean (SD)	Responses, n (%)					
		Strongly disagree	Disagree	Neutral	Agree		Strongly agree
Less stress	4.44 (0.78)	4 (1.1)	3 (0.8)	32 (9)	109 (30.8)		206 (58.2)
Less likely to avoid care	4.37 (0.92)	9 (2.5)	7 (2)	33 (9.3)	100 (28.2)		205 (57.9)
Better able to find and use health care information	4.45 (0.76)	2 (0.6)	3 (0.8)	37 (10.4)	102 (28.8)		210 (59.3)
Better able to find provider	4.51 (0.74)	4 (1.1)	3 (0.8)	20 (5.6)	108 (30.5)		219 (61.9)
More prepared to engage with physician	4.43 (0.78)	3 (0.8)	2 (0.6)	41 (11.6)	102 (28.8)		206 (58.2)

**Table 3.** Unadjusted composite LGBTQ+^a^ Health Care Navigation impact score (n=354).

Composite scores^b^	LGBTQ+ Navigation impact values
Average score, mean (SD)	4.44 (0.69)
Score of less than 4, n (%)	68 (19.2)
Score of 4 or more but less than 5, n (%)	128 (36.2)
Score or 5, n (%)	158 (44.63)

a LGBTQ+: lesbian, gay, bisexual, transgender, queer, and others.

b Composite scores are the unadjusted average of the 5 individual member-reported outcomes for each member.

**Table 4.** Association between LGBTQ+ Health Care Navigation impact scores and case and member characteristics (n=354).

	LGBTQ+ Health care navigation impact score, mean (SD)	*P* value
Region		<.001^b^
Midwest	4.59 (0.52)	
Northeast	4.39 (0.72)	
South	4.69 (0.51)	
West	4.20 (0.79)	
Requester service		.41^c^
Provider request	4.45 (0.67)	
Resources/support	3.6 (1.77)	
Returning user		.054^c^
No	4.36 (0.67)	
Yes	4.50 (0.71)	
Race		.01^b^
Non-White	4.40 (0.74)	
White	4.30 (0.73)	
N/A^d^	4.55 (0.64)	
Ethnicity, n (%)		.008^b^
Non-Hispanic	4.30 (0.74)	
Hispanic	4.46 (0.69)	
N/A	4.55 (0.64)	
Pronouns, n (%)		.02^b^
Single pronouns	4.33 (0.76)	
Multiple pronouns	4.36 (0.51)	
N/A	4.55 (0.64)	
Gender identity, n (%)		.003^b^
N/A	4.55 (0.64)	
Cisgender	4.36 (0.66)	
Multiple gender identities	4.56 (0.37)	
Other^e^	4.25 (0.77)	
Transgender	4.11 (0.95)	
Sexual orientation, n (%)		.05^b^
N/A	4.54 (0.65)	
Bisexual	4.48 (0.59)	
Gay/lesbian	4.31 (0.69)	
Multiple sexual orientations	4.34 (0.68)	
Other^f^	4.27 (0.97)	

a LGBTQ+: lesbian, gay, bisexual, transgender, queer, and others.

b Determined using ANOVA with Tukey post hoc tests.

c Determined using 2-tailed *t* tests.

d N/A: not available.

e Other gender identities included agender, gender fluid, intersex, or nonbinary.

f Other sexual orientations included aromantic, asexual, pansexual, queer, or heterosexual.

## Discussion

### Principal Findings

To our knowledge, this is the first study to empirically measure the impact of a national, virtual LGBTQ+-specialized care navigation service on member-reported outcomes among commercially insured adults. We found the navigation service, which was considered a comprehensive framework to health care access [14], had a meaningful impact on multiple process outcomes that were associated with the use of preventive services, improved health, and lower long-term costs.

While LGBTQ+ individuals are more likely to receive preventive screenings and have better management of mental health conditions when under the care of an identity-affirming provider [33], the challenge lies in finding LGBTQ+ affirming providers [33,34]. Our study highlighted that members who used LGBTQ+ Navigation reported improved confidence in finding identity-affirming providers and reported being prepared to interact with them. Care avoidance is a significant health care concern as well, leading to the reduced use of preventive services, compromised health outcomes, and elevated long-term health care expenses [35,36]. This is particularly pertinent to the LGBTQ+ communities, which are more prone than other populations to avoid seeking care due to barriers and discrimination [37].

Our study found that 86.2% (305/354) of members reported they were less likely to avoid care after using the LGBTQ+ Navigation service. Moreover, LGBTQ+ Navigation increased members’ ability to find, understand, and use health care information, which may lead to potential improvements in medication adherence, increased use of preventive care, reduced emergency room visits, and enhanced management of chronic care [38]. These results echo existing literature discussing the positive impact of LGBTQ+ navigation services on health care access and health care outcomes [28,39,40]. On the other hand, a fifth of members had an LGBTQ+ Navigation impact score that was less than 4, which means they were more likely to respond with neutral, disagree, or strongly disagree to the survey questions. This may indicate the presence of systemic barriers, such as laws, legislation, and insurance policies, that LGBTQ+ Navigation services are unable to address. Further analysis of members who did not respond affirmatively is needed to enhance services and better understand their unmet needs.

We found that care navigation positively impacted all member subgroups, with scores above 4 on the LGBTQ+ Navigation impact, though there were statistically significant differences between the groups. Notably, we found statistically significant differences by gender identity, with the lowest score for transgender individuals. In the context of historical stigma and continual discrimination toward transgender individuals compared to cisgender individuals, the lower scores suggest the need for additional gains toward more inclusive care environments. We also found the highest impact scores were among members who did not provide their demographic information on intake forms. This makes it difficult to interpret differences and highlights an area for future research when more data is available.

Research to date on the impact of care navigation for LGBTQ+ communities has been limited to small samples and focused on subsets of the communities, specifically those receiving care for chronic diseases (eg, HIV) or gender-affirming care [27,28]. This formative evaluation’s results are encouraging, and similar tailored care navigation services have the potential to broaden the reach and scalability of health care access, particularly benefiting LGBTQ+ populations who often face challenges in navigating their health care needs. While promising, we recognize that the observed differences between respondents and nonrespondents could introduce biases into our findings, particularly concerning regional disparities and engagement levels. For instance, even though we observed no differences between respondents and nonrespondents in the use of care navigation services, the participants included in our analyses were less likely to be from the South. Moreover, members with multiple cases were more likely to provide feedback. These patterns suggest that certain groups may face barriers to participation, which could skew the overall results. To address this, we recommend targeted outreach efforts aimed at increasing survey participation among underrepresented groups, particularly those in the South and individuals with fewer cases. By implementing these strategies, we aim to create a more balanced dataset in future research, which would ultimately improve the generalizability and inclusivity of the study’s conclusions.

Overall, continuous evaluation of these services can serve as a supplementary tool for employers seeking to promote health equity and improve belonging among employees. This is particularly important as discrimination and stigma against these communities persist in the United States. Such services may help reduce barriers by lowering out-of-pocket costs, allowing employees to access care without financial strain, while also addressing both insurance policy limitations and state-level policy constraints [28]. Furthermore, they show promise for enhanced health literacy among employees [41], which may empower them to navigate the health care system and make informed decisions about their health. Therefore, scalable and system-level changes that use navigation services are essential to reaching a larger proportion of the LGBTQ+ population.

### Limitations

This study is not without limitations. First, the results may lack generalizability. Included Health was used as a case study, and the members who completed member-reported outcomes differed from those who did not. Second, there may be survey-response bias or a ceiling effect in member-reported outcomes. We recognize that a subset of members provided follow-up feedback through the surveys. Future research examining strategies to encourage increased survey completion rates among members may offer greater insights into patient-reported outcomes related to the Included Health LGBTQ+ Navigation service. Third, while members were asked to report the impact since working with Included Health, member-reported outcomes were solicited at case completion only and do not reflect changes from a presurvey to postsurvey. Fourth, some members had access to additional Included Health services, such as virtual care, which may have influenced the responses to the survey. However, less than 7% of respondents used virtual care services and the survey came directly from their LGBTQ+ care coordinator after their case was closed, limiting the likelihood that the survey captured their experience with other Included Health services. Fifth, we were unable to differentiate the impact of LGBTQ+ Navigation by the multiple race and ethnicity, sexual orientation, and gender identities captured in the intake form due to small sample sizes and the need for mutually exclusive categories to support statistical testing. However, we felt it was important to examine if any differences were present by subgroup definitions with the data available. By consolidating members into broader race, sexual orientation, and gender identity categories, we were able to maximize data availability and better understand the differences between groups. Finally, most members did not provide optional demographic characteristics, which limited our ability to draw conclusions from the subgroup analyses. Moreover, we were unable to distinguish whether the examined case was for the policy member or for their family members since the data was not initially collected for research purposes. Despite these limitations, the strengths of the study include a national sample of members from the LGBTQ+ communities and an initial assessment of the impact of navigation on key process outcomes that can lead to improved health and reduced costs.

### Conclusion

Health care navigation services can offer advocacy and guidance to members by facilitating connections to verified in-network providers, focusing on benefits navigation tailored to LGBTQ+ health needs and providing education and advocacy for both clinical and nonclinical needs. The Included Health LGBTQ+ Navigation service demonstrates potential public health utility and value. Further research is needed to examine the use of care navigation within segments of the LGBTQ+ communities. Moreover, investigating how care navigators addressed specific barriers will inform future navigation opportunities and policy development.

## Supplementary material

10.2196/64137 Multimedia Appendix 1 Original member responses to optional gender identity and sexual orientation fields within member request form (n=176).

10.2196/64137 Checklist 1 STROBE (Strengthening the Reporting of Observational Studies in Epidemiology) checklist of items that should be included in reports of cohort studies.

## Abbreviations

IRB: Institutional Review Board
LGBTQ+: lesbian, gay, bisexual, transgender, queer, and others
STROBE: Strengthening the Reporting of Observational Studies in Epidemiology
WPATH: World Professional Association for Transgender Health
