# Supplementary material for: Impact of a Virtual Care Navigation Service on Member-Reported Outcomes Among Lesbian, Gay, Bisexual, Transgender, and Queer Populations: Case Study
Source: JMIR Form Res. 2025 Jan 9;9:e64137. doi: 10.2196/64137 (PMC11737804; doi:10.2196/64137)
Supplement: Multimedia Appendix 1 [file formative-v9-e64137-s001.docx]

Supplement File 2. Original member responses to optional gender identity and sexual orientation fields within member request form (n=176).

| **Gender Identity** | |
| --- | --- |
| Cisgender | 3 (1.70%) |
| Man | 57 (32.39%) |
| Woman | 18 (10.23%) |
| Transgender | 8 (4.55%) |
| Transman | 11 (6.25%) |
| Transwoman | 24 (13.64%) |
| Transmasculine | 1 (0.57%) |
| Transfeminine | 1 (0.57%) |
| Nonbinary/Nonconforming | 29 (16.48%) |
| Agender | 2 (1.14%) |
| Genderfluid | 6 (3.41%) |
| Questioning | 0 (0%) |
| Other | 2 (1.14%) |
| **Sexual Orientation** | |
| Gay | 73 (41.48%) |
| Lesbian | 19 (10.80%) |
| Bisexual | 42 (23.86%) |
| Queer | 46 (26.14%) |
| Straight | 5 (2.84%) |
| Pansexual | 18 (10.23%) |
| Asexual | 8 (4.55%) |
| Aromantic | 6 (3.41%) |
| Demisexual | 1 (0.57%) |
| Other | 3 (1.70%) |

Note. Non-mutually exclusive categories; participants were allowed to select all that apply. This table does not include the count of missing/null responses.

**Data cleaning for gender identity and sexual orientation.**

Sexual orientation and gender identity response options were not mutually exclusive and changed over time and across forms. Given this, and due to small sample size, we created distinct categories that enabled statistical testing which were informed by the National Academies’ Consensus Report for measuring sexual orientation and gender identity.^22^ The table below presents the final sexual orientation and gender identity categories for analysis and how the response options mapped into these categories. If a member selected multiple options that were mapped to more than one category, they were categorized as ‘multiple select’.

Table 2. Final sexual orientation and gender identity categories to support statistical testing through increased sample sizes and mutually exclusive categories

| **Variable definitions** | **Response options within forms** |
| --- | --- |
| **Sexual orientation** |  |
| Lesbian or Gay | lesbian; gay |
| Bisexual | bisexual |
| Other | aromantic; asexual; pansexual; queer; heterosexual; free text option was not bisexual, lesbian, or gay |
| Multiple select | Selected one or more sexual orientations of Lesbian or Gay; Bisexual; and Other. |
| **Gender identity** |  |
| Cisgender | cisgender man; cisgender woman |
| Transgender | transgender man; transgender woman; transgender |
| Other | agender; genderfluid; intersex; nonbinary; free text option did not indicate cisgender or transgender |
| Multiple select | Selected one or more gender identities of Cisgender; Transgender; or Other. |
